# Supplementary material for: The mTOR pathway genes MTOR, Rheb, Depdc5, Pten, and Tsc1 have convergent and divergent impacts on cortical neuron development and function
Source: eLife. 2024 Feb 27;12:RP91010. doi: 10.7554/eLife.91010 (PMC10942629; doi:10.7554/eLife.91010)
Supplement: Figure 1—source data 1. [file elife-91010-fig1-data1.docx]

**Figure 1–source data 1: Summary statistics for Figure 1**

| **Fig. 1d: p-S6 INTENSITY** | | | | | |  |  |  |  |  | |
| --- | --- | --- | --- | --- | --- | --- | --- | --- | --- | --- | --- |
|  |  |  |  | **Nested one-way ANOVA^a^** | |  |  |  |  | **Nested one-way ANOVA^a^** | |
|  | **Control** | ***Rheb^Y35L^*** | ***MTOR^S2215Y^*** | **F, DFn, DFd** | **p-value** | **Control** | ***Depdc5^KO^*** | ***Pten^KO^*** | ***Tsc1^KO^*** | **F, DFn, DFd** | **p-value** |
| **Mean**  **± SD** | 1.00  ± 0.7 | 2.50  ± 0.7 | 1.84  ± 0.6 | 36.46, 2, 12 | <0.0001 | 1.00  ± 0.5 | 2.04  ± 0.8 | 2.52  ± 0.5 | 2.17  ± 0.8 | 25.31, 3, 19 | <0.0001 |
| **No. of animals** | 5 | 4 | 6 |  |  | 8 | 5 | 6 | 4 |  |  |
| **No. cells/ animal** | 15 | 15 (6 for one mouse) | 15 |  |  | 15 | 15 | 15 (8, 11 for two mice) | 15 |  |  |
| **Total cells** | 75 | 51 | 90 |  |  | 120 | 75 | 79 | 60 |  |  |
| **Fig. 1e: NEURON SOMA SIZE** | | | | | |  |  |  |  |  | |
|  |  |  |  | **Nested one-way ANOVA^a^** | |  |  |  |  | **Nested one-way ANOVA^a^** | |
|  | **Control** | ***Rheb^Y35L^*** | ***MTOR^S2215Y^*** | **F, DFn, DFd** | **p-value** | **Control** | ***Depdc5^KO^*** | ***Pten^KO^*** | ***Tsc1^KO^*** | **F, DFn, DFd** | **p-value** |
| **Mean**  **± SD** | 84.3  ± 15.9 | 283.2  ± 86.4 | 294.8  ± 80.0 | 57.17, 2, 12 | <0.0001 | 105.0  ± 21.0 | 174.7  ± 55.8 | 285.2  ± 90.8 | 222.6  ± 87.3 | 49.30, 3, 19 | <0.0001 |
| **No. of animals** | 5 | 4 | 6 |  |  | 8 | 5 | 6 | 4 |  |  |
| **No. cells/ animal** | 15 | 15 (6 for one mouse) | 15 |  |  | 15 | 15 | 15 (8, 11 for two mice) | 15 |  |  |
| **Total cells** | 75 | 51 | 90 |  |  | 120 | 75 | 79 | 60 |  |  |
| **Fig. 1g: NEURON POSITIONING (% cells in layer 2/3)** | | | | | |  |  |  |  |  | |
|  |  |  |  | **One-way ANOVA** | |  |  |  |  | **One-way ANOVA** | |
|  | **Control** | ***Rheb^Y35L^*** | ***MTOR^S2215Y^*** | **F (DFn, DFd)** | **p-value** | **Control** | ***Depdc5^KO^*** | ***Pten^KO^*** | ***Tsc1^KO^*** | **F (DFn, DFd)** | **p-value** |
| **Mean ± SD** | 82.7  ± 10.2 | 19.2  ± 12.6 | 31.6  ± 7.4 | F (2, 11) = 55.09 | <0.0001 | 87.6  ± 5.3 | 54.8  ± 11.7 | 80.6  ± 11.3 | 40.9  ± 10.8 | F (3, 16) = 22.81 | <0.0001 |
| **No. of animals** | 5 | 3 | 6 |  |  | 7 | 4 | 6 | 3 |  |  |
| **Fig. 1h: NEURON POSITIONING (% cells in bins)** | | | | | |  |  |  |  |  | |
|  | **No. of animals** | **Two-way repeated measures ANOVA** | | | |  | **No. of animals** | **Two-way repeated measures ANOVA** | | | |
|  |  |  | **F (DFn, DFd)** | | **p value** |  |  |  | **F (DFn, DFd)** | | **p value** |
| **Control** | 5 | **Bin x Group** | F (18, 99) = 12.58 | | <0.0001 | **Control** | 7 | **Bin x Group** | F (27, 144) = 5.802 | | <0.0001 |
| ***Rheb^Y35L^*** | 3 | **Bin** | F (9, 99) = 16.14 | | <0.0001 | ***Depdc5^KO^*** | 4 | **Bin** | F (9, 144) = 95.04 | | <0.0001 |
| ***MTOR^S2215Y^*** | 6 | **Group** | F (2, 11) = 0.3745 | | 0.6961 | ***Pten^KO^*** | 6 | **Group** | F (3, 16) = 1.021 | | 0.4095 |
|  |  |  |  |  |  | ***Tsc1^KO^*** | 3 |  |  |  |  |

^a^The nested one-way ANOVA fits a mixed-effects model wherein the main factor is treated as a fixed factor and the nested factor is treated as a random factor.

^b^Post-hoc analyses were performed using Holm-Šídák multiple comparison test. Significant post-hoc results (p<0.05) are denoted with symbols (*, #, Ɏ) on the graphs, with the number of symbols 1-4 denoting the significant levels p<0.05, <0.01, <0.001, and <0.0001, respectively. For all two-way repeated measured and mixed-effects model ANOVA, all significant results (p<0.05) are denoted with one symbol regardless of the significant level for clearness on the graphs.
