# Supplementary material for: The mTOR pathway genes MTOR, Rheb, Depdc5, Pten, and Tsc1 have convergent and divergent impacts on cortical neuron development and function
Source: eLife. 2024 Feb 27;12:RP91010. doi: 10.7554/eLife.91010 (PMC10942629; doi:10.7554/eLife.91010)
Supplement: Figure 1—figure supplement 2—source data 1. [file elife-91010-fig1-figsupp2-data1.docx]

**Figure 1–source data 2: Summary statistics for Figure 1-figure supplement 2**

| **Fig 1-fig. supplement 2b: NEURON SOMA SIZE** **(P7-9)** | | | | | |  |  |  |  |  | |
| --- | --- | --- | --- | --- | --- | --- | --- | --- | --- | --- | --- |
|  |  |  | **Nested t-test^a^** | | |  |  |  |  | **Nested one-way ANOVA^a^ *** | |
|  | **Control** | ***MTOR^S2215Y^*** | **t, df** | **F, DFn, DFd** | **p-value** | **Control** | ***Depdc5^KO^*** | ***Pten^KO^*** | ***Tsc1^KO^*** | **F, DFn, DFd** | **p-value** |
| **Mean ± SD** | 76.2 ± 1.7 | 144.4 ±2.9 | 13.22, 4 | 174.8, 1, 4 | 0.0002 | 75.9 ± 1.5 | 100.1 ± 2.1 | 151.7 ± 2.8 | 116.7 ± 2.1 | 26.87, 3, 18 | <0.0001 |
| **No. of animals** | 3 | 3 |  |  |  | 4 | 4 | 6 | 8 |  |  |
| **No. cells/ animal** | 30 | 30 |  |  |  | 30 | 30 | 30 | 30 |  |  |
| **Total cells** | 90 | 90 |  |  |  | 120 | 120 | 180 | 240 |  |  |

^a^The nested t-test and ANOVA fit a mixed-effects model wherein the main factor is treated as a fixed factor and the nested factor is treated as a random factor.

^b^Post-hoc analyses were performed using Holm-Šídák multiple comparison test. Significant post-hoc results (p<0.05) are denoted with symbols (*) on the graphs, with the number of symbols 1-4 denoting the significant levels p<0.05, <0.01, <0.001, and <0.0001, respectively.
