# Supplementary material for: The mTOR pathway genes MTOR, Rheb, Depdc5, Pten, and Tsc1 have convergent and divergent impacts on cortical neuron development and function
Source: eLife. 2024 Feb 27;12:RP91010. doi: 10.7554/eLife.91010 (PMC10942629; doi:10.7554/eLife.91010)
Supplement: Figure 2—source data 1. [file elife-91010-fig2-data1.docx]

**Figure 2–source data 1: Summary statistics for Figure 2**

| **Fig. 2b: MEMBRANE CAPACITANCE (pF)** | | | | | |  |  |  |  |  | |
| --- | --- | --- | --- | --- | --- | --- | --- | --- | --- | --- | --- |
|  |  |  |  | **Nested one-way ANOVA^a^** | |  |  |  |  | **Nested one-way ANOVA^a^** | |
|  | **Control** | ***Rheb^Y35L^*** | ***MTOR^S2215Y^*** | **F, DFn, DFd** | **p-value** | **Control** | ***Depdc5^KO^*** | ***Pten^KO^*** | ***Tsc1^KO^*** | **F, DFn, DFd** | **p-value** |
| **Mean**  **± SD** | 121.6  ± 36.6 | 402.3  ± 105.4 | 462.4  ± 147.0 | 109.6, 2, 20 | <0.0001 | -130.8 ± 38.1 | 205.0 ± 69.4 | 340.0 ± 142.5 | 295.9 ± 109.2 | 37.77, 3, 127 | <0.0001 |
| **No. of animals** | 10 | 5 | 8 |  |  | 10 | 5 | 6 | 8 |  |  |
| **No. cells/ animal** | 1-7 | 4-8 | 1-7 |  |  | 3-8 | 4-8 | 1-8 | 1-5 |  |  |
| **Total cells** | 43 | 25 | 35 |  |  | 48 | 28 | 28 | 27 |  |  |
| **Fig. 2c: MEMBRANE CONDUCTANCE (nS)** | | | | | |  |  |  |  |  | |
|  |  |  |  | **Nested one-way ANOVA^a^** | |  |  |  |  | **Nested one-way ANOVA^a^** | |
|  | **Control** | ***Rheb^Y35L^*** | ***MTOR^S2215Y^*** | **F, DFn, DFd** | **p-value** | **Control** | ***Depdc5^KO^*** | ***Pten^KO^*** | ***Tsc1^KO^*** | **F, DFn, DFd** | **p-value** |
| **Mean**  **± SD** | 10.6  ± 3.8 | 20.1  ± 5.3 | 23.7  ± 7.4 | 33.58, 2, 18 | <0.0001 | 8.0  ± 2.4 | 13.1  ± 4.5 | 18.6  ± 9.2 | 13.8  ± 4.1 | 17.83, 3, 25 | <0.0001 |
| **No. of animals** | 8 | 5 | 8 |  |  | 10 | 5 | 6 | 8 |  |  |
| **No. cells/ animal** | 3-6 | 4-8 | 1-6 |  |  | 3-8 | 4-8 | 1-8 | 1-5 |  |  |
| **Total cells** | 34 | 24 | 30 |  |  | 47 | 27 | 27 | 28 |  |  |
| **Fig. 2d: RMP (mV)** | | | | | |  |  |  |  |  | |
|  |  |  |  | **Nested one-way ANOVA^a^** | |  |  |  |  | **Nested one-way ANOVA^a^** | |
|  | **Control** | ***Rheb^Y35L^*** | ***MTOR^S2215Y^*** | **F, DFn, DFd** | **p-value** | **Control** | ***Depdc5^KO^*** | ***Pten^KO^*** | ***Tsc1^KO^*** | **F, DFn, DFd** | **p-value** |
| **Mean**  **± SD** | -74.5  ± 3.8 | -69.0  ± 5.1 | -64.9  ± 5.3 | 24.20, 2, 20 | <0.0001 | -69.7  ± 5.0 | -70.0  ± 5.3 | -67.6  ± 5.3 | -64.7  ± 7.3 | 4.292, 3, 25 | 0.0142 |
| **No. of animals** | 10 | 5 | 8 |  |  | 10 | 5 | 6 | 8 |  |  |
| **No. cells/ animal** | 1-7 | 4-8 | 1-7 |  |  | 3-8 | 4-8 | 1-8 | 1-5 |  |  |
| **Total cells** | 42 | 25 | 36 |  |  | 52 | 28 | 28 | 31 |  |  |
| **Fig. 2f: AP INPUT-OUTPUT CURVE** | | | | | |  |  |  |  |  | |
|  | **No. of neurons** | **Mixed-effects ANOVA** | | | |  | **No. of neurons** | **Mixed-effects ANOVA** | | | |
|  |  | Fixed effects (type III) | **F (DFn, DFd)** | | **p-value** |  |  | Fixed effects (type III) | **F (DFn, DFd)** | | **p-value** |
| **Control** | 36 | **Current step** | F (20, 1657) = 263.4 | | <0.0001 | **Control** | 50 | **Current step** | F (20, 2579) = 600.7 | | <0.0001 |
| ***Rheb^Y35L^*** | 22 | **Group** | F (2, 83) = 39.60 | | <0.0001 | ***Depdc5^KO^*** | 27 | **Group** | F (3, 129) = 22.84 | | <0.0001 |
| ***MTOR^S2215Y^*** | 28 | **Current step x group** | F (40, 1657) = 22.84 | | <0.0001 | ***Pten^KO^*** | 28 | **Current step x group** | F (60, 2579) = 14.66 | | <0.0001 |
|  |  |  |  |  |  | ***Tsc1^KO^*** | 28 |  |  |  |  |
| **Fig. 2g: RHEBOBASE (pA)** | | | | | |  |  |  |  |  | |
|  |  |  |  | **Nested one-way ANOVA^a^** | |  |  |  |  | **Nested one-way ANOVA^a^** | |
|  | **Control** | ***Rheb^Y35L^*** | ***MTOR^S2215Y^*** | **F, DFn, DFd** | **p-value** | **Control** | ***Depdc5^KO^*** | ***Pten^KO^*** | ***Tsc1^KO^*** | **F, DFn, DFd** | **p-value** |
| **Mean**  **± SD** | 544.4  ± 225.3 | 1356.0  ± 634.6 | 1422.0  ± 624.2 | 26.74, 2, 77 | <0.0001 | 484.4  ± 180.1 | 737.3  ± 315.2 | 1071.0  ± 503.5 | 935.7  ± 444.4 | 16.84, 3, 25 | <0.0001 |
| **No. of animals** | 7 | 5 | 6 |  |  | 10 | 5 | 6 | 8 |  |  |
| **No. cells/ animal** | 4-6 | 4-7 | 1-7 |  |  | 3-7 | 4-7 | 1-8 | 1-5 |  |  |
| **Total cells** | 32 | 24 | 24 |  |  | 50 | 27 | 28 | 28 |  |  |
| **Fig. 2h: 1^st^ ISI (ms)** | | | | | | | | | | | |
|  |  |  |  | **Nested one-way ANOVA^a^** | |  |  |  |  | **Nested one-way ANOVA^a^** |  |
|  | **Control** | ***Rheb^Y35L^*** | ***MTOR^S2215Y^*** | **F, DFn, DFd** | **p-value** | **Control** | ***Depdc5^KO^*** | ***Pten^KO^*** | ***Tsc1^KO^*** | **F, DFn, DFd** | **p-value** |
| **Mean**  **± SD** | 15.9 ± 6.4 | 28.7 ± 9.8 | 24.3 ± 10.8 | 9.386, 2, 18 | 0.0016 | 15.5 ± 7.0 | 22.7 ± 9.1 | 12.2 ± 8.0 | 23.1 ± 10.3 | 8.105, 3, 24 | 0.0007 |
| **No. of animals** | 9 | 5 | 7 |  |  | 10 | 5 | 5 | 8 |  |  |
| **No. cells/ animal** | 1-6 | 1-4 | 1-7 |  |  | 3-7 | 4-7 | 2-5 | 2-4 |  |  |
| **Total cells** | 36 | 16 | 25 |  |  | 50 | 27 | 18 | 23 |  |  |
| **Fig. 2i: AP THRESHOLD (mV)** | | | | | |  |  |  |  |  | |
|  |  |  |  | **Nested one-way ANOVA^a^** | |  |  |  |  | **Nested one-way ANOVA^a^** | |
|  | **Control** | ***Rheb^Y35L^*** | ***MTOR^S2215Y^*** | **F, DFn, DFd** | **p-value** | **Control** | ***Depdc5^KO^*** | ***Pten^KO^*** | ***Tsc1^KO^*** | **F, DFn, DFd** | **p-value** |
| **Mean**  **± SD** | -39.2  ± 6.3 | -44.0  ± 12.3 | -41.0  ± 8.4 | 1.937, 2, 78 | 0.1511 | -35.8  ± 9.4 | -39.0  ± 7.9 | -40.0  ± 14.4 | -37.9  ± 11.1 | 0.6587, 3, 25 | 0.5852 |
| **No. of animals** | 7 | 5 | 6 |  |  | 10 | 5 | 6 | 8 |  |  |
| **No. cells/ animal** | 4-6 | 3-7 | 1-6 |  |  | 2-6 | 3-7 | 1-7 | 1-5 |  |  |
| **Total cells** | 33 | 23 | 25 |  |  | 42 | 25 | 26 | 25 |  |  |
| **Fig. 2j: AP PEAK AMPLITUDE (mV)** | | | | | |  |  |  |  |  | |
|  |  |  |  | **Nested one-way ANOVA^a^** | |  |  |  |  | **Nested one-way ANOVA^a^** | |
|  | **Control** | ***Rheb^Y35L^*** | ***MTOR^S2215Y^*** | **F, DFn, DFd** | **p-value** | **Control** | ***Depdc5^KO^*** | ***Pten^KO^*** | ***Tsc1^KO^*** | **F, DFn, DFd** | **p-value** |
| **Mean**  **± SD** | 138.1 ± 10.1 | 139.0 ± 11.6 | 120.3  ± 8.9 | 20.51, 2, 15 | <0.0001 | 135.0  ± 9.1 | 136.1  ± 12.9 | 138.3  ± 21.1 | 126.1  ± 7.8 | 4.239, 3, 116 | 0.0070 |
| **No. of animals** | 7 | 5 | 6 |  |  | 10 | 5 | 6 | 8 |  |  |
| **No. cells/ animal** | 4-6 | 3-7 | 1-6 |  |  | 2-6 | 3-7 | 1-7 | 1-5 |  |  |
| **Total cells** | 33 | 23 | 25 |  |  | 43 | 25 | 27 | 25 |  |  |
| **Fig. 2k: AP HALF-WIDTH (ms)** | | | | | |  |  |  |  |  | |
|  |  |  |  | **Nested one-way ANOVA^a^** | |  |  |  |  | **Nested one-way ANOVA^a^** | |
|  | **Control** | ***Rheb^Y35L^*** | ***MTOR^S2215Y^*** | **F, DFn, DFd** | **p-value** | **Control** | ***Depdc5^KO^*** | ***Pten^KO^*** | ***Tsc1^KO^*** | **F, DFn, DFd** | **p-value** |
| **Mean**  **± SD** | 1.79  ± 0.30 | 1.50  ± 0.18 | 1.44  ± 0.14 | 18.45, 2, 15 | <0.0001 | 1.76  ± 0.43 | 1.65  ± 0.23 | 1.44  ± 0.24 | 1.52  ± 0.23 | 3.679, 3, 25 | 0.0254 |
| **No. of animals** | 7 | 5 | 6 |  |  | 10 | 5 | 6 | 8 |  |  |
| **No. cells/ animal** | 4-6 | 3-7 | 1-6 |  |  | 2-6 | 3-7 | 1-7 | 1-5 |  |  |
| **Total cells** | 33 | 23 | 25 |  |  | 43 | 25 | 27 | 25 |  |  |

^a^The nested one-way ANOVA fits a mixed-effects model wherein the main factor is treated as a fixed factor and the nested factor is treated as a random factor.

^b^Post-hoc analyses were performed using Holm-Šídák multiple comparison test. Significant post-hoc results (p<0.05) are denoted with symbols (*, #, Ɏ) on the graphs, with the number of symbols 1-4 denoting the significant levels p<0.05, <0.01, <0.001, and <0.0001, respectively. For all two-way repeated measured and mixed-effects model ANOVA, all significant results (p<0.05) are denoted with one symbol regardless of the significant level for clearness on the graphs.
