# Supplementary material for: The mTOR pathway genes MTOR, Rheb, Depdc5, Pten, and Tsc1 have convergent and divergent impacts on cortical neuron development and function
Source: eLife. 2024 Feb 27;12:RP91010. doi: 10.7554/eLife.91010 (PMC10942629; doi:10.7554/eLife.91010)
Supplement: Figure 3—source data 1. [file elife-91010-fig3-data1.docx]

**Figure 3–source data 1: Summary statistics for Figure 3**

| **Fig. 3b: HCN4 INTENSITY** | | | | | |  |  |  |  |  | |
| --- | --- | --- | --- | --- | --- | --- | --- | --- | --- | --- | --- |
|  |  |  |  | **Nested one-way ANOVA^a^** | |  |  |  |  | **Nested one-way ANOVA^a^** | |
|  | **Control** | ***Rheb^Y35L^*** | ***MTOR^S2215Y^*** | **F, DFn, DFd** | **p-value** | **Control** | ***Depdc5^KO^*** | ***Pten^KO^*** | ***Tsc1^KO^*** | **F, DFn, DFd** | **p-value** |
| **Mean**  **± SD** | 1.00  ± 0.16 | 1.30  ± 0.25 | 1.36  ± 0.31 | 7.499, 2, 12 | 0.0077 | 1.00  ± 0.16 | 1.26  ± 0.36 | 1.24  ± 0.18 | 1.22  ± 0.20 | 6.579, 3, 18 | 0.0034 |
| **No. of animals** | 5 | 4 | 6 |  |  | 8 | 5 | 5 | 4 |  |  |
| **No. cells/ animal** | 15 (10 for one mouse) | 15 (4 for one mouse, 11 for one mouse) | 15 (14 for on mouse) |  |  | 15 (13 for one mouse) | 15 (12 for two mice, 14 for one mouse) | 15 (7 for one mouse) | 15 (8 for one mouse) |  |  |
| **Total cells** | 70 | 45 | 89 |  |  | 118 | 68 | 67 | 53 |  |  |
| **Fig. 3d: IV CURVE** | | | | | |  |  |  |  |  | |
|  | **No. of neurons** | **Mixed-effects ANOVA** | | | |  | **No. of neurons** | **Mixed-effects ANOVA** | | | |
|  |  | Fixed effects (type III) | **F (DFn, DFd)** | | **p-value** |  |  | Fixed effects (type III) | **F (DFn, DFd)** | | **p-value** |
| **Control** | 42 | **Voltage step** | F (6, 537) = 821.1 | | <0.0001 | **Control** | 47 | **Voltage step** | F (6, 714) = 569.5 | | <0.0001 |
| ***Rheb^Y35L^*** | 24 | **Group** | F (2, 95) = 144.5 | | <0.0001 | ***Depdc5^KO^*** | 28 | **Group** | F (3, 126) = 20.08 | | <0.0001 |
| ***MTOR^S2215Y^*** | 32 | **Voltage step x group** | F (12, 537) = 92.68 | | <0.0001 | ***Pten^KO^*** | 27 | **Voltage step x group** | F (18, 714) = 22.98 | | <0.0001 |
|  |  |  |  |  |  | ***Tsc1^KO^*** | 28 |  |  |  |  |
| **Fig. 3e:** Δ**IV CURVE** | | | | | |  |  |  |  |  | |
|  | **No. of neurons** | **Mixed-effects ANOVA** | | | |  | **No. of neurons** | **Mixed-effects ANOVA** | | | |
|  |  | Fixed effects (type III) | **F (DFn, DFd)** | | **p-value** |  |  | Fixed effects (type III) | **F (DFn, DFd)** | | **p-value** |
| **Control** | 42 | **Voltage step** | F (6, 537) = 83.01 | | <0.0001 | **Control** | 47 | **Voltage step** | F (6, 720) = 78.95 | | <0.0001 |
| ***Rheb^Y35L^*** | 24 | **Group** | F (2, 95) = 61.77 | | <0.0001 | ***Depdc5^KO^*** | 28 | **Group** | F (3, 126) = 16.35 | | <0.0001 |
| ***MTOR^S2215Y^*** | 32 | **Voltage step x group** | F (12, 537) = 33.33 | | <0.0001 | ***Pten^KO^*** | 27 | **Voltage step x group** | F (18, 720) = 5.506 | | <0.0001 |
|  |  |  |  |  |  | ***Tsc1^KO^*** | 28 |  |  |  |  |
| **Fig. 3f: I_h_ AMPLITUDE at -90 mV (pA)** | | | | | |  |  |  |  |  | |
|  |  |  |  | **Nested one-way ANOVA^a^** | |  |  |  |  | **Nested one-way ANOVA^a^** | |
|  | **Control** | ***Rheb^Y35L^*** | ***MTOR^S2215Y^*** | **F, DFn, DFd** | **p-value** | **Control** | ***Depdc5^KO^*** | ***Pten^KO^*** | ***Tsc1^KO^*** | **F, DFn, DFd** | **p-value** |
| **Mean**  **± SD** | -4.4  ± 7.2 | -53.1  ± 56.7 | -212.9  ± 151.4 | 29.22, 2, 20 | <0.0001 | -8.7  ± 12.1 | -18.6  ± 23.7 | -28.0  ± 41.1 | -51.2  ± 31.2 | 10.34, 3, 25 | <0.0001 |
| **No. of animals** | 10 | 5 | 8 |  |  | 10 | 5 | 6 | 8 |  |  |
| **No. cells/ animal** | 1-7 | 4-8 | 1-7 |  |  | 3-8 | 4-8 | 1-8 | 1-5 |  |  |
| **Total cells** | 42 | 24 | 36 |  |  | 47 | 28 | 27 | 29 |  |  |
| **Fig. 3h: IV CURVE (ZATERBADINE, *MTOR^S2215Y^*)** | | | | | |  |  |  |  |  | |
|  | **No. of neurons** | **Two-way repeated measures ANOVA** | | | |  |  |  | | | |
|  |  |  | **F (DFn, DFd)** | | **p value** |  |  |  |  | |  |
| **Pre-zatebradine** | 4 | **Voltage step** | F (6, 18) = 19.45 | | <0.0001 |  |  |  |  | |  |
| **Post-zatebradine** | 4 | **Group** | F (1, 3) = 58.36 | | 0.0047 |  |  |  |  | |  |
|  |  | **Voltage step x group** | F (6, 18) = 27.54 | | <0.0001 |  |  |  |  | |  |
| **Fig. 3i:** Δ**IV CURVE (ZATEBRADINE, *MTOR^S22515Y^*)** | | | | | |  |  |  |  |  | |
|  | **No. of neurons** | **Two-way repeated measures ANOVA** | | | |  |  |  | | | |
|  |  |  | **F (DFn, DFd)** | | **p value** |  |  |  |  | |  |
| **Pre-zatebradine** | 4 | **Voltage step** | F (6, 18) = 17.70 | | <0.0001 |  |  |  |  | |  |
| **Post-zatebradine** | 4 | **Group** | F (1, 3) = 49.57 | | =0.0059 |  |  |  |  | |  |
|  |  | **Voltage step x group** | F (6, 18) = 13.65 | | <0.0001 |  |  |  |  | |  |
| **Fig. 3l: RMP (mV; ZATERBADINE, *control*, *MTOR^S22515Y^*)** | | | | | |  |  |  |  |  | |
|  | **No. of neurons** | **Two-way repeated measures ANOVA** | | | |  |  |  | | | |
|  |  |  | **F (DFn, DFd)** | | **p value** |  |  |  |  | |  |
| **Pre-zatebradine (control)** | 6 | **Treatment** | F (1, 9) = 37.42 | | 0.0002 |  |  |  |  | |  |
| **Post-zatebradine (control)** | 6 | **Group** | F (1, 9) = 2.747 | | 0.1318 |  |  |  |  | |  |
| **Pre-zatebradine (*MTOR^S22515Y^*)** | 4 | **Treatment x group** | F (1, 9) = 33.83 | | 0.0003 |  |  |  |  | |  |
| **Post-zatebradine (*MTOR^S22515Y^*)** | 4 |  |  | |  |  |  |  |  | |  |

^a^The nested one-way ANOVA fits a mixed-effects model wherein the main factor is treated as a fixed factor and the nested factor is treated as a random factor.

^b^Post-hoc analyses were performed using Holm-Šídák multiple comparison test. Significant post-hoc results (p<0.05) are denoted with symbols (*, #, Ɏ) on the graphs, with the number of symbols 1-4 denoting the significant levels p<0.05, <0.01, <0.001, and <0.0001, respectively. For all two-way repeated measured and mixed-effects model ANOVA, all significant results (p<0.05) are denoted with one symbol regardless of the significant level for clearness on the graphs.
