# Supplementary material for: The mTOR pathway genes MTOR, Rheb, Depdc5, Pten, and Tsc1 have convergent and divergent impacts on cortical neuron development and function
Source: eLife. 2024 Feb 27;12:RP91010. doi: 10.7554/eLife.91010 (PMC10942629; doi:10.7554/eLife.91010)
Supplement: Figure 3—figure supplement 3—source data 1. [file elife-91010-fig3-figsupp3-data1.docx]

**Figure 3–source data 2: Summary statistics for Figure 3-figure supplement 3**

| **Fig. 3-fig. supplement 3b: IV CURVE (BaCl_2_)** | | | | |  |  |  |  |  | |
| --- | --- | --- | --- | --- | --- | --- | --- | --- | --- | --- |
|  | **No. of neurons** | **Mixed-effects ANOVA** | | |  |  |  | | | |
|  |  | Fixed effects  (type III) | **F (DFn, DFd)** | **p-value** |  |  |  |  | |  |
| **Pre-BaCl_2_ (control)** | 10 | **Voltage step** | F (8, 72) = 201.6 | <0.0001 |  |  |  |  | |  |
| **Post-BaCl_2_ (control)** | 10 | **Group** | F (1, 9) = 46.18 | <0.0001 |  |  |  |  | |  |
|  |  | **Voltage step x group** | F (8, 55) = 45.52 | <0.0001 |  |  |  |  | |  |
| **Pre BaCl_2_ (*Tsc1^KO^*)** | 13 | **Voltage step** | F (8, 96) = 81.41 | <0.0001 |  |  |  |  | |  |
| **Post-BaCl_2_ (*Tsc1^KO^*)** | 13 | **Group** | F (1, 12) = 12.46 | 0.0041 |  |  |  |  | |  |
|  |  | **Voltage step x group** | F (8, 64) = 20.46 | <0.0001 |  |  |  |  | |  |
| **Fig. 3-fig. supplement 3c:** Δ**IV CURVE (BaCl_2_)** | | | | |  |  |  |  |  | |
|  | **No. of neurons** | **Mixed-effects ANOVA** | | |  |  |  | | | |
|  |  | Fixed effects  (type III) | **F (DFn, DFd)** | **p-value** |  |  |  |  | |  |
| **Pre-BaCl_2_ (control)** | 10 | **Voltage step** | F (8, 72) = 4.337 | 0.0003 |  |  |  |  | |  |
| **Post-BaCl_2_ (control)** | 10 | **Group** | F (1, 9) = 2.310 | 0.1629 |  |  |  |  | |  |
|  |  | **Voltage step x group** | F (8, 56) = 0.3825 | 0.9255 |  |  |  |  | |  |
| **Pre BaCl_2_ (*Tsc1^KO^*)** | 13 | **Voltage step** | F (8, 96) = 12.53 | <0.0001 |  |  |  |  | |  |
| **Post-BaCl_2_ (*Tsc1^KO^*)** | 13 | **Group** | F (1, 12) = 0.7970 | 0.3895 |  |  |  |  | |  |
|  |  | **Voltage step x group** | F (8, 64) = 2.306 | 0.0307 |  |  |  |  | |  |
| **Fig. 3-fig. supplement 3e: IV CURVE (BaCl_2_-sensitive current)** | | | | |  |  |  |  |  | |
|  | **No. of neurons** | **Mixed-effects ANOVA** | | |  |  |  | | | |
|  |  | Fixed effects  (type III) | **F (DFn, DFd)** | **p-value** |  |  |  |  | |  |
| **Control** | 10 | **Voltage step** | F (1.252, 19.41) = 92.62 | <0.0001 |  |  |  |  | |  |
| ***Tsc1^KO^*** | 13 | **Group** | F (1, 21) = 1.036 | 0.3204 |  |  |  |  | |  |
|  |  | **Voltage step x group** | F (8, 124) = 7.724 | <0.0001 |  |  |  |  | |  |
| **Fig. 3-fig. supplement 3f: CONDUCTANCE (nS, BaCl_2_)** | | | | |  |  |  |  |  | |
|  | **No. of neurons** | **Two-way repeated measures ANOVA** | | |  |  |  | | | |
|  |  |  | **F (DFn, DFd)** | **p value** |  |  |  |  | |  |
| **Control** | 10 | **Group** | F (1, 20) = 20.04 | 0.0002 |  |  |  |  | |  |
| ***Tsc1^KO^*** | 12 | **Treatment** | F (1, 20) = 82.05 | <0.0001 |  |  |  |  | |  |
|  |  | **Group x treatment** | F (1, 20) = 0.0002453 | 0.9877 |  |  |  |  | |  |
| **Fig. 3-fig. supplement 3g: CONDUCTANCE (% change, BaCl_2_)** | | | | |  |  |  |  |  | |
|  | **No. of neurons** | **Unpaired t-test** | | |  |  |  | | | |
|  |  | t, df | **p-value** |  |  |  |  |  | |  |
| **Control** | 10 | t=3.841, df=20 | 0.0010 |  |  |  |  |  | |  |
| ***Tsc1^KO^*** | 12 |  |  |  |  |  |  |  | |  |
| **Fig. Fig. 3-fig. supplement 3h: RMP (mV, BaCl_2_)** | | | | |  |  |  |  |  | |
|  | **No. of neurons** | **Two-way repeated measures ANOVA** | | |  |  |  | | | |
|  |  |  | **F (DFn, DFd)** | **p value** |  |  |  |  | |  |
| **Control** | 10 | **Group** | F (1, 21) = 0.2692 | 0.6093 |  |  |  |  | |  |
| ***Tsc1^KO^*** | 13 | **Treatment** | F (1, 21) = 134.7 | 0.0001 |  |  |  |  | |  |
|  |  | **Group x treatment** | F (1, 21) = 9.281 | 0.0061 |  |  |  |  | |  |
| **Fig. 3-fig. supplement 3i: RMP (% change, BaCl_2_)** | | | | |  |  |  |  |  | |
|  | **No. of neurons** | **Unpaired t-test** | | |  |  |  | | | |
|  |  | t, df | **p-value** |  |  |  |  |  | |  |
| **Control** | 10 | t=2.810, df=21 | 0.0105 |  |  |  |  |  | |  |
| ***Tsc1^KO^*** | 13 |  |  |  |  |  |  |  | |  |

^b^Post-hoc analyses were performed using Holm-Šídák multiple comparison test. Significant post-hoc results (p<0.05) are denoted with symbols (*) on the graphs, with the number of symbols 1-4 denoting the significant levels p<0.05, <0.01, <0.001, and <0.0001, respectively.
