# Supplementary material for: The mTOR pathway genes MTOR, Rheb, Depdc5, Pten, and Tsc1 have convergent and divergent impacts on cortical neuron development and function
Source: eLife. 2024 Feb 27;12:RP91010. doi: 10.7554/eLife.91010 (PMC10942629; doi:10.7554/eLife.91010)
Supplement: Figure 4—source data 1. [file elife-91010-fig4-data1.docx]

**Figure 4 – source data 1: Summary statistics for Figure 4**

| **Fig. 4b: sEPSC FREQUENCY (Hz)** | | | | | |  |  |  |  |  | |
| --- | --- | --- | --- | --- | --- | --- | --- | --- | --- | --- | --- |
|  |  |  |  | **Nested one-way ANOVA^a^** | |  |  |  |  | **Nested one-way ANOVA^a^** | |
|  | **Control** | ***Rheb^Y35L^*** | ***MTOR^S2215Y^*** | **F, DFn, DFd** | **p-value** | **Control** | ***Depdc5^KO^*** | ***Pten^KO^*** | ***Tsc1^KO^*** | **F, DFn, DFd** | **p-value** |
| **Mean ± SD** | 4.4 ± 1.7 | 3.3 ± 0.8 | 4.7 ± 2.1 | 2.550, 2, 15 | 0.1114 | 3.9 ± 1.4 | 5.1 ± 1.7 | 4.8 ± 1.5 | 5.3 ± 1.9 | 4.054, 3, 89 | 0.0095 |
| **No. of animals** | 7 | 5 | 6 |  |  | 9 | 5 | 5 | 8 |  |  |
| **No. cells/ animal** | 3-6 | 4-5 | 2-7 |  |  | 2-5 | 2-6 | 3-4 | 1-4 |  |  |
| **Total cells** | 33 | 21 | 25 |  |  | 34 | 20 | 17 | 22 |  |  |
| **Fig. 4c: sEPSC AMPLITUDE (pA)** | | | | | |  |  |  |  |  | |
|  |  |  |  | **Nested one-way ANOVA^a^** | |  |  |  |  | **Nested one-way ANOVA^a^** | |
|  | **Control** | ***Rheb^Y35L^*** | ***MTOR^S2215Y^*** | **F, DFn, DFd** | **p-value** | **Control** | ***Depdc5^KO^*** | ***Pten^KO^*** | ***Tsc1^KO^*** | **F, DFn, DFd** | **p-value** |
| **Mean ± SD** | -8.1 ± 2.1 | -11.5 ± 2.5 | -12.6 ± 3.0 | 19.20, 2, 15 | <0.0001 | -6.7 ± 1.3 | -8.7 ± 1.9 | -10.1 ± 4.1 | -8.5 ± 1.8 | 4.280, 3, 23 | 0.0154 |
| **No. of animals** | 7 | 5 | 6 |  |  | 9 | 5 | 5 | 8 |  |  |
| **No. cells/ animal** | 3-6 | 4-5 | 2-7 |  |  | 2-5 | 2-6 | 3-4 | 1-4 |  |  |
| **Total cells** | 33 | 21 | 25 |  |  | 34 | 20 | 17 | 22 |  |  |
| **Fig. 4d: sEPSC TOTAL CHARGE (pA/ms)** | | | | | |  |  |  |  |  | |
|  |  |  |  | **Nested one-way ANOVA^a^** | |  |  |  |  | **Nested one-way ANOVA^a^** | |
|  | **Control** | ***Rheb^Y35L^*** | ***MTOR^S2215Y^*** | **F, DFn, DFd** | **p-value** | **Control** | ***Depdc5^KO^*** | ***Pten^KO^*** | ***Tsc1^KO^*** | **F, DFn, DFd** | **p-value** |
| **Mean ± SD** | 0.20 ± 0.12 | 0.26 ± 0.08 | 0.43 ± 0.22 | 8.300, 2, 15 | 0.0037 | 0.20 ± 0.10 | 0.35 ± 0.14 | 0.41 ± 0.21 | 0.38 ± 0.20 | 9.273, 3, 89 | <0.0001 |
| **No. of animals** | 7 | 5 | 6 |  |  | 9 | 5 | 5 | 8 |  |  |
| **No. cells/ animal** | 3-6 | 4-5 | 2-7 |  |  | 2-5 | 2-6 | 3-4 | 1-4 |  |  |
| **Total cells** | 33 | 21 | 25 |  |  | 34 | 20 | 17 | 22 |  |  |

^a^The nested one-way ANOVA fits a mixed-effects model wherein the main factor is treated as a fixed factor and the nested factor is treated as a random factor.

^b^Post-hoc analyses were performed using Holm-Šídák multiple comparison test. Significant post-hoc results (p<0.05) are denoted with symbols (*, #, Ɏ) on the graphs, with the number of symbols 1-4 denoting the significant levels p<0.05, <0.01, <0.001, and <0.0001, respectively. For all two-way repeated measured and mixed-effects model ANOVA, all significant results (p<0.05) are denoted with one symbol regardless of the significant level for clearness on the graphs.
